# Effects of oral/enteral nutrition alone versus plus pantoprazole on gastrointestinal bleeding in critically ill patients with low risk factor: a multicenter, randomized controlled trial

**DOI:** 10.3906/sag-1911-42

**Published:** 2020-06-23

**Authors:** Kürşat GÜNDOĞAN, Emre KARAKOÇ, Turgut TEKE, Avşar ZERMAN, Aliye ESMAOĞLU, Şahin TEMEL, Muhammet GÜVEN, Murat SUNGUR

**Affiliations:** 1 Division of Intensive Care, Department of Internal Medicine, Faculty of Medicine, Erciyes University, Kayseri Turkey; 2 Division of Intensive Care, Department of Internal Medicine, Faculty of Medicine, Çukurova University, Adana Turkey; 3 Division of Intensive Care, Department of Pulmonary Disease, Faculty of Medicine, Necmettin Erbakan University, Konya Turkey; 4 Intensive Care Unit, Department of Internal Medicine, Ministry of Health Adana Numune Training and Educational Hospital, Adana Turkey; 5 Division of Intensive Care, Department of Anesthesiology and Reanimation, Faculty of Medicine, Erciyes University, Kayseri Turkey

**Keywords:** Critical illness, enteral nutrition, pantoprazole, gastrointestinal bleeding, stress ulcer

## Abstract

**Background/aim:**

Critically ill patients are at risk of developing gastrointestinal (GI) bleeding due to stress causing mucosal damage. Aim of the study was to determine the effect of oral/enteral nutrition with or without concomitant pantoprazole on upper GI bleeding in low risk critically ill patients.

**Materials and methods:**

This was a prospective, randomized, open-label, multicenter study conducted with intensive care unit (ICU) patients receiving oral/enteral nutritional support. Patients were randomly assigned into two groups including intervention group (received oral/EN plus pantoprazole) and control group (received only oral/EN).

**Results:**

A total of 300 patients (intervention group: 152, control group: 148) participated in the study. Overall, 226 (75%) patients were fed by orally and 74 (25%) patients fed by enteral tube feeding. Median duration of nutritional support 4 (range: 2–33) days. Overt upper GI bleeding was noted only in one patient (0.65%) who was in the intervention group. The overall length of ICU stay of 4 (2–105) days, while ICU stay was significantly longer in the intervention group than in the control group (P = 0.006).

**Conclusions:**

Our findings seems to indicate that in patients who are at low risk for GI bleeding and under oral/enteral nutritional support, the use of PPIs may not reduce the risk of bleeding, however these results are imprecise because of low event (GI bleeding) rate and limited power.

## 1. Introduction

Gastrointestinal (GI) bleeding secondary to stress-related mucosal lesions are considered likely to be encountered in critically ill patients as associated with increased risk of mortality and morbidity in an intensive care unit (ICU). Mucosal erosions on the gastric luminal surface occur in approximately 75%–100% of ICU patients within the first 24 h of admission [1,2]. These erosions often cause bleeding by penetrating the superficial capillaries. Stress-related GI bleeding occurs in less than 5% of the ICU patients [3–7]. Enteral nutrition (EN) has been considered to have protective effects against the bleeding of stress ulcers by neutralizing the acidic pH level in the gastric lumen which contributes to structural and functional integrity of the mucosal surface along with trophic effects on the GI mucosa [8–10]. There is insufficient evidence regarding the association between EN and stress ulcer hemorrhage in critically ill patients, and therefore it becomes challenging for clinicians to make suggestions. Major risk factors for stress ulcer hemorrhage are mechanical ventilation (MV), coagulopathy and burns [3,11]. Proton pump inhibitors (PPI) and histamine receptor blockers (H2RB) are the main drugs used for the prophylaxis of stress ulcer related GI bleeding. Studies have shown that 90% of patients admitted to ICU receive prophylaxis for stress ulcer related GI bleeding [12, 13]. However, drugs (H2RB, PPI) used for prophylaxis against stress ulcer related GI bleeding have some undesirable effects in critically ill population. These drugs, which suppress gastric acid secretion, can cause hospital-associated pneumonia and Clostridium difficile enterocolitis [14–16].

Most of previous studies about stress ulcer prophylaxis belong to 1980s and early 1990s [3,10,11]. Most of the patients recruited in these studies received nothing per oral (NPO) and EN was not a widely practiced nutritional support method by clinicians in those years. In some studies, use of EN was reported to be associated with lesser likelihood of less GI bleeding development in patients with stress ulcers [10,11]. In a limited number of animal studies, enteral feeding was shown to protect against stress-related gastric mucosal damage [8,9,17].

Among critically ill adults receiving EN, we have hypothesized that those patients not treated with pantoprazole will not have an increased risk of overt GI bleeding compared to pantoprazole-treated patients. 

This study was designed to comparatively evaluate upper GI bleeding due to stress ulcers in low risk critically ill patients receiving oral/enteral nutrition support with or without concomitant pantoprazole therapy. 

## 2. Materials and methods

This multicenter, prospective, controlled, randomized, open-label trial was performed in five different ICU clinics between August 2016 and August 2017. Erciyes University Ethics Committee approved the study (Date of Approval: 06/05/2016, Protocol No: 2016/289). Written informed consent was obtained from each patient or their legal representatives prior to the start of the study. 

During the study period, all eligible patients were screened for inclusion in the study. Patients aged ≥18 years who were expected to stay in ICU for >24 h and had no contraindications to EN within the first 24 h of ICU admission were included in the study. Evidence of active GI bleeding during current hospitalization prior to study enrollment, presence of coagulopathy (PLT < 50.000/mm3, INR > 1.5, aPTT > 2 x control), acid suppressing treatment prior to admission, pregnancy or lactation, gastric ulcer (history or documented), burns involving >30% body surface area, head injury or increased intracranial pressure, partial or complete gastrectomy, shock, multisystem trauma, exposure to gastric irritant drugs and lack of informed consent were the exclusion criteria of the study. 

### 2.1. Randomization

We conducted a randomized, parallel group, multicenter study. The patients were randomly assigned to receive oral/enteral nutrition plus pantoprazole (40 mg IV or oral, ones daily) (Intervention group) or oral/enteral nutrition alone (Control group). Randomization was stratified based on the APACHE II scores calculated before randomization, to enable similar disease severity in both groups. Randomization was performed by the research nurse by using previously prepared closed and opaque envelopes. When there is another patient with similar APACHE II score, that patient was randomized to the opposite group with the previous patient. 

The selection of the EN formula was at physicians’ discretion, and included Nutrison Diason®, Nutrision Protein Plus Multi Fiber®, Pulmocare®, Isosource Protein®, Jevity, Isosource Protein®, Impact Glutamin®, Novasource GI Control®, Glucerna Select® or Nepro HP®.

Oral supplements were also administered according to the clinician’s decisions and included Ensure plus Fiber and Resource Energy.

Initial patient data were collected at the time of randomization. Demographic data of the patients, primary complaint for ICU admission, time from ICU admission to study enrollment and onset of nutritional therapy were also recorded. APACHE II, modified NUTRIC score and GCS were calculated within the first 24 h of admission. Nutritional therapy was performed according to ESPEN and ASPEN critical care nutrition guidelines [18–20 ]. All patients were screened on a daily basis for overt and significant GI bleeding. In addition, the need for invasive or noninvasive MV was also monitored and recorded on a daily basis. Daily follow-up continued for each patient until transfer from ICU, occurrence of any contraindication to oral/enteral nutrition or death. SOFA score of patients was recorded daily as well as the length of ICU stay and mortality in the ICU.

### 2.2. Outcome measures

Primary outcome: Patients were followed from the study enrolment to the ICU discharge (4 weeks) or the cessation of EN for possible GI bleeding. Overt GI bleeding was considered as the presence of coffee ground-like emesis, hematemesis, melena or hematochezia. Significant GI bleeding was defined by 3-point decrease in hematocrit levels within 24 h as accompanied by overt GI bleeding or by an unexplained 6-point decrease in hematocrit during a 48-h period [21,22].

Secondary outcomes were daily SOFA scores, length of ICU stay, length of invasive or noninvasive MV and ICU mortality rates.

### 2.3. Statistical analysis

All statistical analyses were made by SPSS 22.0 (IBM Corp., Armonk, NY, USA). Data are expressed as the mean ± standard deviation (SD) or the median (lower and upper quartiles). Comparisons between groups for continuous variables were performed using the Student t-Test (normal distribution) or the Mann-Whitney U test (nonnormal distribution). The χ2 test was used to analyze categorical variables. A P value of <0.05 was considered statistically significant.

## 3. Results

**Figure 1:**
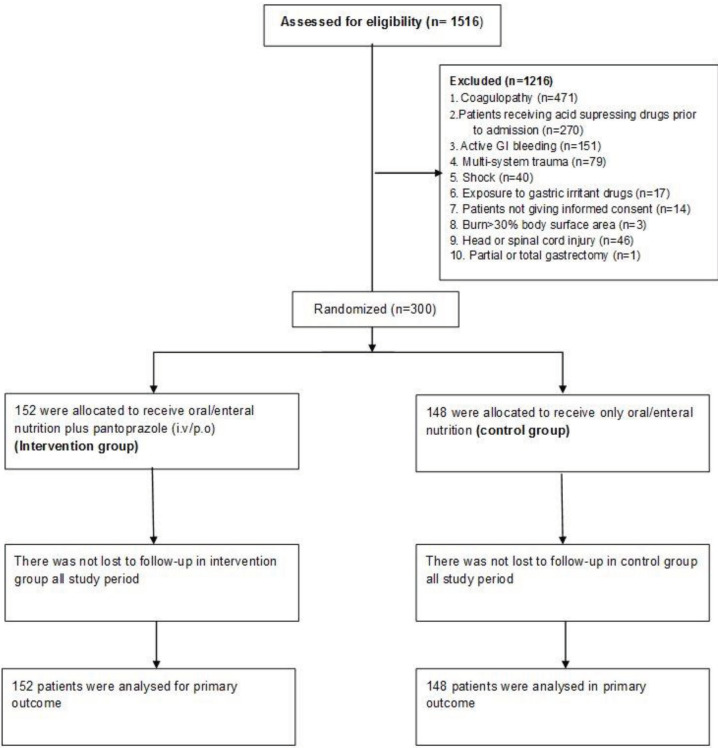
Flow chart.Enrolled patients are as follows: ........ University Medical ICU: 118 patients, ........ University Anesthesiology ICU: 26 patients, ............ University Medical ICU: 67 patients, .......... University Pulmonary ICU: 57 patients, Ministry of Health .................... Hospital ICU: 32 patients.

Of 1516 patients assessed for eligibility, 1216 patients were excluded and 300 patients who met inclusion criteria were included in the study, as randomized to intervention (n = 152) and control (n = 148) groups (Figure 1). 

Demographic characteristics of patients are provided in Table 1. The overall age of the patients was 64 ± 18 years. The most common cause of ICU admission was respiratory failure and noted in 148 (49%) patients, as followed by postoperative conditions in 42 (14%) patients and neurologic disorders in 33 (11%) patients. The APACHE II score was 19 ± 6 in the overall study population, and similar between the intervention and control groups (19 ± 6 versus 19 ± 6, P > 0.05). First day mean GCS was 13 ± 3. Mean NUTRIC score was 4 ± 2. Baseline mean hemoglobin value was 10.7 ± 2.2 g/dL, platelet count was 235000±122000/mm3, international normalized value (INR) was 1.2 ± 0.2 and aPTT was 35 ± 17 s in the overall study population.

**Table 1:** Baseline patient characteristics.

Variables	Intervention group N = 152	Control group N = 148	P
Age ± SD, years	65 ± 17	63 ± 19	0.484
Sex, n (%) Male Female	81 (53) 71 (47)	89 (60) 59 (40)	0.232
APACHE II score ±SD	19 ± 6	19 ± 6	0.686
GCS (First day), ±SD	13 ± 3	12 ± 4	0.260
Modified NUTRIC score ±SD	4 ± 2	4 ± 2	0.991
BMI ± SD	27 ± 6	25 ± 6	0.044
Reason for ICU admission, n (%) Respiratory failure Postoperative patients Neurologic disorders Sepsis/septic shock Renal failure (acute/chronic) Cardiac arrest/other cardiac disorders Intoxication Hepatic failure (acute/chronic) Other	82 (54) 20 (15) 11 (7) 13 (9) 14 (9) 4 (2.6) 3 (2) 1 (0.7) 4 (2.6)	66 (45) 22 (15) 22 (15) 16 (11) 12 (8) 5 (3.4) 2 (1.4) 1 (0.7) 2 (1.4)	0.549
Hemoglobin ±SD (g/dL)	10.8 ± 2.2	10.5 ± 2.1	0.277
Platelet counts ±SD	234 ± 125	237 ± 119	0.837
INR ± SD	1.2 ± 0.2	1.2 ± 0.1	0.543
PTT ± SD	36 ± 19	34 ± 15	0.271
Time to study enrolment (range) (h)	14 (2–24)	14 (1–29)	0.043
Time to nutritional intervention (range) (h)	6 (1–24)	5 (1–22)	0.304
Vasopressor therapy, n (%)	4 (2.6)	3 (2.0)	0.729
Noninvasive MV, n (%)	27 (18)	14 (10)	0.036
Invasive MV, n (%)	23 (15)	21 (14)	0.076
Calculated target calorie, ±SD(kcal)	1487 ± 235	1464 ± 233	0.666
Type of nutritional support, n (%) Oral Enteral	119 (78) 33 (22)	107 (72) 41 (28)	0.229

APACHE II: Acute physiology and chronic health evaluation II, GCS: Glasgow coma score, BMI: Body mass index, ICU: Intensive care unit INR: International normalized ratio, PTT: Partial thromboplastin time, MV: Mechanical ventilation.

The median time to start oral/enteral nutrition after admission to the ICU was 6 (1–24) h. The median time from ICU admission to study enrollment was 14 (1–29) h (Table 1). The mean calculated target calorie of the patients was 1470 ± 260 kcal/day. Overall, 75% of patients received oral nutritional supplements (ONS), while EN was administered via tube feeding (ETF) in 25% of the patients. ETF was applied by gastric route in 48 (60%) and by postpyloric route in 32 (40%) patients (Table 1).

Standard oxygen therapy was applied in 63 patients, while 85 patients required invasive and noninvasive MV. Noninvasive MV was needed in 14% of the patients and invasive MV was used in 15% of the patients (Table 1). Seven (2.3%) patients required vasopressor therapy during ICU stay. Pantoprazole was given intravenously in 113 (74%) and orally in 39 (26%) patients in the intervention group. Pantoprazole was started at a median 4 (range 0–24) h after admission to the ICU. PPI was given to intervention group during ICU stay. Any patient who can receive PPI orally received the treatment per oral.

### 3.1. Outcomes

**Figure 2:**
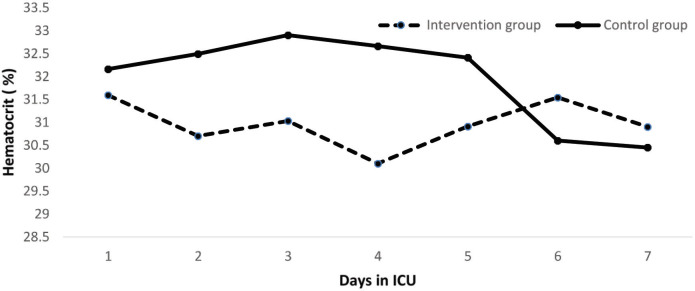
Hematocrit profile for the first 7 days in intervention and control groups. Study group patient’s hematocrit profile (%) (First day: 32.2 ± 7.1, second day: 32.5 ± 7.0, third day: 32.9 ± 6.8, fourth day: 32.6 ± 7.5, fifth day: 32.4 ± 8.1, sixth day: 30.6 ± 7.4 and seventh day: 30.4 ± 6.3). Control group patient’s hematocrit profile (%) (First day: 31.6 ± 5.6, second day: 30.7 ± 5.4, third day: 31.0 ± 6.7, fourth day: 30.1 ± 5.3, fifth day: 30.9 ± 4.9, sixth day: 31.5 ± 4.5 and seventh day: 30.9 ± 3.8).

Primary outcomes: Overt GI bleeding was observed in one patient in oral/enteral nutrition plus pantoprazole group (0.65%). This patient was discharged from the study at the 48th h of the study due to hemodynamic instability. There was no upper GI bleeding among patients who received only oral/enteral nutrition (Table 2). The seven-day hematocrit profile of all patients were as follows; first day: 31.8 ± 6.4, second day: 31.7 ± 6.4, third day: 32.2 ± 6.8, fourth day: 31.7 ± 6.8, fifth day: 31.8 ± 7.1, sixth day: 30.9 ± 6.5 and seventh day: 30.6 ± 5.3. There was no hematocrit decrease to indicate significant upper GI bleeding in both groups (Figure 2).

**Table 2:** Patient’s primary and secondary outcomes.

Outcomes	Intervention group N = 152	Control group N = 148	p
Overt GI bleeding, n (%)	1 (0.65)	0 (0)	-
SOFA scores, (range) First day Third day Fifth day Seventh day	4.00 (1–12) 3.00 (1–11) 4.00 (0–13) 4.00 (2–12)	3.00 (0–11) 4.00 (2–11) 4.00 (1–11) 4.00 (2–9)	0.824 0.053 0.548 0.865
Amount of calorie received, ±SD(kcal)	1458 ± 317	1478 ± 264	0.562
Duration of nutritional support, days (range)	4 (2–33)	3 (2–12)	0.004
Length of ICU stay, days (range)	5 (2–36)	3 (2–105)	0.006
Duration of invasive MV, days (range)	4 (1–30)	3 (1–9)	0.780
Duration of noninvasive MV, days (range)	1 (1–3)	2 (1–3)	0.275
ICU mortality, n (%)	24 (15)	19 (12)	0.103

GI: Gastrointestinal, MV: Mechanical ventilation, ICU: Intensive care unit.

Secondary outcomes: Overall, SOFA scores were 4 (1–2) on day 1, 3 (1–11) on day 3, 4 (0–13) on day 5 and 4 (2–12) on day 7. No significant difference was noted between study groups in terms daily SOFA scores (P > 0.05) (Table 2).

The mean calorie goal reached at the end of 24 h was 1468 ± 292 kcal/day with no significant difference in amount of calories received by the end 24th h between study groups (P = 0.562) (Table 2).

The length of nutrition was median 4 (range: 2–33) days overall, while patients in the intervention group received significantly longer nutritional support as compared with control group patients (P = 0.004). Nutritional intervention was temporarily discontinued in 43 patients during ICU follow up, while this temporary NPO period was due to procedures in the ICU in 39 patients and based on clinician’s decision in 4 patients. 

The length of ICU stay was median 4 (range: 2–105) days in the overall study population, while the intervention group was associated with significantly longer ICU stay as compared with the control group patients (P = 0.006). 

The duration of invasive MV was 3 (range: 1–30) days and that of noninvasive MV was 1 (range: 1–6) day, with no significant difference in duration of invasive and noninvasive MV between the study groups (P > 0.05). The ICU mortality rate was 14% and similar between intervention and control groups. 

## 4. Discussion

This prospective, multicenter, randomized controlled open-label trial showed that there was no statistical difference for upper GI bleeding of stress ulcers among critically ill patients receiving oral/enteral feeding alone or together with pantoprazole for stress ulcer bleeding prophylaxis.

Stress ulcer prophylaxis therapy has been routinely used for the past 3 decades in ICUs. In 1994, Cook et al. performed a study with 2250 critically ill patients and concluded coagulopathy and respiratory failure to be independent risk factors for stress ulcer related upper GI bleeding [3]. Stress ulcer bleeding prophylaxis has become almost a routine therapy in these patients preceding this study.

Gross gastric lesions were reported to be visible within the first 72 h following the endoscopy procedure in 75%–100% of critically ill patients [2]. In previous animal and human studies, EN therapy has been shown to reduce splanchnic blood flow, GI motility, increase gastric pH levels and reduce stress-related mucosal related complications [8,10,17,23,24]. In some studies performed in 1980’s, EN was reported to be protective against stress ulcer bleeding in respiratory failure and burn patients. However, being small scale studies without randomization, these studies failed to draw adequate attention to stress ulcer bleeding [10,11]. Currently there are some randomized studies indicated that EN may provide stress ulcer bleeding prophylaxis. These studies are discussed below. 

The effects of early EN on prophylaxis for stress ulcer related bleeding were investigated in a randomized, controlled, double blind trial in mechanically ventilated critically ill patients. These patients were randomized to either early EN and placebo or EN and intravenous pantoprazole groups. Both groups were followed for clinically significant or overt GI bleeding. A total of 102 patients were recruited and 55 patients were given EN plus pantoprazole, whereas 47 patients received EN and placebo. Two (1.96%) overt GI bleeding cases were observed during the study period; one from each group [4].

In another prospective, a double blind, randomized controlled trial in a mixed ICU where the patients that received EN and MV were also included in the study, the benefit and harm associated with the administration of pantoprazole were evaluated [5]. Intravenous pantoprazole was administered to the patients in the study group and placebo was administered to the patients in the control group. Major outcomes from the study included clinically significant GI bleeding, VAP and *Clostridium difficile* infection whereas the minor outcomes included overt bleeding and mortality. A total of 214 patients (106 patients in group 1 and 108 patients in group 2) were enrolled in the study. There was no clinical significant GI bleeding in either group. There were 9 patients with overt GI bleeding; 3 (2.8%) patients with overt GI bleeding in the pantoprazole group and 6 (5.6%) patients in placebo group [5].

Both of these randomized controlled studies had similar patient characteristics with the current study. The main similarity between the studies seems to be the protective role of EN against stress ulcer related bleeding.

The efficacy and safety of withholding PPIs in critical ill patients were investigated in a pilot randomized controlled study by Canadian Critical Care Trials Group (REVISE study) [25]. The patients were recruited from 10 different ICUs in Canada, Saudi Arabia and Australia. The study included patients that were mechanically ventilated for at least 48 h. The study included a total of 91 patients; 49 patients received intravenous pantoprazole and 42 placebo. Upper GI bleeding was observed in 6.1% of the pantoprazole group and 4.8% of placebo group (P = 1.0). Most patients (89%) did not receive EN during the first three days of study enrollment. Mean APACHE II scores were 21 and similar in both groups [25]. The mean APACHE II score was 19 in our study along with lower GI bleeding rate, which may be explained by the differences in critical level of ICU patients.

In another study regarding follow up for potential GI bleeding among 200 surgical trauma ICU patients (73.5% were TBI), pharmacologic stress ulcer prophylaxis was discontinued once EN provided full caloric requirements for patients requiring MV [6]. Authors noted only one upper bleeding in the patients receiving full EN [6]. Notably our findings also revealed only one upper GI bleeding under EN support, while multisystem, TBI and spinal trauma patients were excluded from the current study

In our study, patients with a multisystem trauma, TBI, spinal trauma, burns, refractory shock and coagulopathy were excluded, while patients with respiratory failure were included in accordance with study by Cook et al. indicated respiratory failure to be an independent risk factor for upper GI bleeding. Respiratory failure was the primary diagnosis in 148 (49 %) of our patients and 85 (28%) of them required invasive or noninvasive MV support. Among the patients, there was only one patient with upper GI bleeding. Our study and the abovementioned studies have indicated that stress ulcer prophylaxis may not be required in critically ill patients, with respiratory failure without coagulopathy and refractory shock, receiving oral/enteral nutrition therapy. 

The effect of prophylaxis for GI bleeding in the ICU was studied by Kraig et al. in 3298 adult patients who were randomly assigned to receive daily, single-bolus, intravenous pantoprazole (40 mg) or placebo during their nonselective ICU stay [7]. There was no significant difference between the pantoprazole group and the placebo group in mortality rates by 90 days after randomization (31.1% and 30.4%, respectively). There was no difference in the secondary outcome of clinically important GI bleeding in pantoprazole and control groups (2.5%, 4.2%, P = 0.58 respectively). EN was started in 58.2% of the patients in pantoprazole group and 56.4% in control group, on the first day of the study. EN rate was 85.8% in pantoprazole group and 85.3% in control group, on the fifth day of the study. Their analyses were not stratified according to EN administration, which could have modified the outcomes.

It was stated that additional data are needed to determine the clinical effects of prophylaxis for GI bleeding in the ICU to quantify any protective or harmful effects attributable to the coadministration of EN in the editorial for this study [26].

In the current study, ICU stay was significantly longer in the intervention group than in the control group. Albeit not significant statistically, a tendency for higher mortality rate was noted in the intervention group. The reason for these negative outcomes in intervention group may be related to the pneumonia and *C. difficile *infection which may be caused by pantoprazole. Unfortunately, we did not record these outcomes in our study. We reported this as a major limitation of our study. 

Oral/enteral nutrition was initiated after median 6 h of ICU admission and the target calorie goal was reached by the 24th h in 90% of the patients. This may explain the very low number of upper GI bleeding cases in our study.

The main limitations of the current study seem to be the relatively small sample size and lack of data on acquired pneumonia and *C. difficile* infection due to unavailability of related hospital records. This is not a blinded study and there was no placebo group which might have also affected the results. We did not perform power analysis to detect necessary number of the patients before the study, which is another major limitation. 

In conclusion, this multicenter prospective randomized open-label study evaluated effects of oral/enteral nutrition plus pantoprazole or oral/enteral nutrition alone, in critically ill patients with low risk factor for GI bleeding. Both groups consisted of critically ill patients who were fed via enteral route. Our findings emphasize the likelihood of no need for GI bleeding prophylaxis among low risk critically ill patients receiving oral/enteral nutrition. 

## Acknowledgments/Disclaimers

We thank NESTLE company for supporting the study with providing research nurse staff in study sites. NESTLE was not involved in the study hypothesis/design, execution, analysis, or interpretation.

This research did not receive any specific grant from funding agencies in the public, commercial, or not-for-profit sectors.

## Author statement

K. Gündoğan, M. Güven, E. Karakoç and M. Sungur equally contributed to the conception and design of the research; K. Gündoğan, M.Güven, S.Temel and M. Sungur contributed to the interpretation of the data; and K. Gündoğan, A. Zerman, T. Teke, A. Esmaoğlu, S. Temel and M. Sungur drafted the manuscript. All authors critically revised the manuscript, agree to be fully accountable for ensuring the integrity and accuracy of the work, and read and approved the final manuscript.
